# Gender Bias in Artificial Intelligence: Severity Prediction at an Early Stage of COVID-19

**DOI:** 10.3389/fphys.2021.778720

**Published:** 2021-11-29

**Authors:** Heewon Chung, Chul Park, Wu Seong Kang, Jinseok Lee

**Affiliations:** ^1^Department of Biomedical Engineering, College of Electronics and Information, Kyung Hee University, Yongin-si, South Korea; ^2^Department of Internal Medicine, Wonkwang University School of Medicine, Iksan, South Korea; ^3^Department of Trauma Surgery, Cheju Halla General Hospital, Jeju-si, South Korea

**Keywords:** COVID-19, severity prediction, artificial intelligence bias, gender dependent bias, feature importance

## Abstract

Artificial intelligence (AI) technologies have been applied in various medical domains to predict patient outcomes with high accuracy. As AI becomes more widely adopted, the problem of model bias is increasingly apparent. In this study, we investigate the model bias that can occur when training a model using datasets for only one particular gender and aim to present new insights into the bias issue. For the investigation, we considered an AI model that predicts severity at an early stage based on the medical records of coronavirus disease (COVID-19) patients. For 5,601 confirmed COVID-19 patients, we used 37 medical records, namely, basic patient information, physical index, initial examination findings, clinical findings, comorbidity diseases, and general blood test results at an early stage. To investigate the gender-based AI model bias, we trained and evaluated two separate models—one that was trained using only the male group, and the other using only the female group. When the model trained by the male-group data was applied to the female testing data, the overall accuracy decreased—sensitivity from 0.93 to 0.86, specificity from 0.92 to 0.86, accuracy from 0.92 to 0.86, balanced accuracy from 0.93 to 0.86, and area under the curve (AUC) from 0.97 to 0.94. Similarly, when the model trained by the female-group data was applied to the male testing data, once again, the overall accuracy decreased—sensitivity from 0.97 to 0.90, specificity from 0.96 to 0.91, accuracy from 0.96 to 0.91, balanced accuracy from 0.96 to 0.90, and AUC from 0.97 to 0.95. Furthermore, when we evaluated each gender-dependent model with the test data from the same gender used for training, the resultant accuracy was also lower than that from the unbiased model.

## Introduction

As artificial intelligence (AI) becomes more widely adopted, the problem of model bias has become increasingly apparent. Bias has long been a critical area of research and concern in AI, and it reflects widespread societal biases about race, gender, biological gender, age, and culture (Ntoutsi et al., 2020; Kapur, 2021; Kim et al., 2021; Tubadji et al., 2021). In this study, we investigated the bias from gender-dependent AI models and aimed to present new insights into the model bias issue. For the investigation, we extended our previous AI study, which is to predict patient severity in the early stage of coronavirus disease (COVID-19) (Chung et al., 2021).

The COVID-19 pandemic has had a major effect on healthcare systems globally. Since early 2020, it has spread rapidly worldwide, exceeding 200 million cases and 4.5 million deaths (Honein et al., 2020). Until recently, medical experts expected cases and deaths to decrease by increasing the administered vaccine doses to 5 billion, but the numbers are still increasing. In a COVID-19 pandemic situation, the most critical issue in managing COVID-19 patients is to triage patients at high risk of mortality and provide tailored treatment, such that medical costs and mortality rates can be reduced. To predict disease severity in COVID-19 patients, numerous AI models have been proposed (Altschul et al., 2020; Zhu et al., 2020; Lessmann et al., 2021; Paiva Proença Lobo Lopes et al., 2021; Shan et al., 2021; Yaşar et al., 2021). We recently developed an AI model that predicts severity based on data from 5,601 COVID-19 patients from all national and regional hospitals across South Korea, as of April 2020 (Chung et al., 2021). Clinical severity was categorized into low and high groups. The low-severity group corresponded to unaffected activity and the presence of oxygen support with nasal prong or facial mask and non-invasive ventilation; meanwhile, the high-severity group corresponded to invasive ventilation, multiorgan failure (requiring extracorporeal membrane oxygenation), and death. For the AI model input, we used 37 medical records, namely, basic patient information, physical index, initial examination findings, clinical findings, comorbidity diseases, and general blood test results at an early stage.

In this study, for the bias issue investigation, we trained the two separate AI models based on each gender (male and female) using the same data (Chung et al., 2021). We then evaluated and compared the performances (1). We tested the AI model trained with male data using female data, and (2) then, tested the AI model trained with female data using male data. We also compared the performance of the unbiased AI model trained using all data, regardless of gender, and proved the superiority of the unbiased model over the biased model. Furthermore, we compared the performances of the biased and unbiased models when testing with the test data from the same gender used for training in the biased model. Based on the result comparison, we further discussed limiting the diversity of even the data of the specific group on which the model was trained.

## Materials and Methods

### Datasets

This study was approved by the Korea Disease Control and Prevention Agency (KDCA) and the Wonkwang University Hospital. The requirement for informed consent was waived. For 5,628 patients, confirmed to have COVID-19 as of April 2020, 37 medical records were available, namely, basic patient information, physical index, initial examination findings, clinical findings, comorbidity diseases, and general blood test results at an early stage. Out of 5,628 COVID-19 patient records, the clinical severity information was missing in 27 patient records, so we excluded them from our study. Thus, we used 5,601 patient data records to develop the AI prediction model for clinical severity. Table 1 summarizes the detailed medical records used to develop the AI model for severity prediction. The clinical severity was categorized into low and high groups. The low-severity group had no reduction in activity and was provided oxygen support *via* nasal prong or facial mask or provided non-invasive ventilation; meanwhile, the high-severity group corresponded to invasive ventilation, multiorgan failure (requiring extracorporeal membrane oxygenation), or death. Supplementary Table 1 summarizes the statistical values of medical records according to low- and high-severity groups. Regarding gender, the numbers of men in the low- and high-severity groups were 2,166 and 144, respectively. The numbers of women in the low- and high-severity groups were 3,164 and 127, respectively.

**TABLE 1:** Medical records used in developing AI model for severity prediction.

**Index**	**Items**	**Data**
1	Basic patient information	Age
2		Gender
3		Pregnancy
4		Pregnancy week
5	Physical index	Body mass index
6	Initial examination findings	Systolic blood pressure
7		Diastolic blood pressure
8		Heart rate
9		Temperature
10	Clinical findings	Fever
11		Cough
12		Sputum production
13		Sore throat
14		Runny nose/rhinorrhea
15		Muscle aches/myalgia
16		Fatigue/malaise
17		Shortness of breath/dyspnea
18		Headache
19		Altered consciousness/confusion
20		Vomiting/nausea
21		Diarrhea
22	Current or previous comorbidity diseases	Diabetes mellitus
23		Hypertension
24		Heart failure
25		Chronic cardiac disease
26		Asthma
27		Chronic obstructive pulmonary disease
28		Chronic kidney disease
29		Cancer
30		Chronic liver disease
31		Rheumatism/autoimmune diseases
32		Dementia
33	General blood test results	Hemoglobin
34		Hematocrit
35		Lymphocyte
36		Platelets
37		White blood cell

*AI, artificial intelligence.*

### Data Split for Training and Testing

Table 2 summarizes the training and testing data. We split the data into training (80%) and testing (20%) datasets in a stratified manner concerning severity. Specifically, for the male group (*n* = 2,310), we split the data groups into 1,848 training and 462 testing data. In the training data, the numbers of low- and high-severity groups were 1,732 and 116, respectively. In the testing data, the number of low- and high-severity groups was 434 and 28, respectively. For the female group (*n* = 3,291), we split the data groups into 2,632 training and 659 testing data. In the training data, the number of low- and high-severity groups was 2,535 and 97, respectively. In the testing data, the number of low- and high-severity groups was 629 and 30, respectively.

**TABLE 2:** Number of data groups for training and testing based on gender and severity.

**Dataset**		**Low-severity group**	**High-severity group**	**Total**
Training data	Male	1,732	116	1,848
	Female	2,535	97	2,632
Testing data	Male	434	28	462
	Female	629	30	659
Total		5,330	271	5,601

### Data Augmentation and K-Fold Cross-Validation

For each gender, the training dataset (1,848 men and 2,633 women) was randomly shuffled and partitioned into five equal folds in a stratified manner. For the male-dependent AI model, each fold included 347 low-severity and 23 high-severity records. For the female-dependent AI model, each fold included 506 low-severity and 20 high-severity records. Of the 5-folds, a single fold was retained as the validation dataset for testing the model, and the remaining 4-folds were used as the training data. We repeated the process 10 times, with each of the 5-folds used exactly once as validation data. Here, because the number of low-severity records was much higher than that of high-severity records, we upsampled the high-severity data using the synthetic minority oversampling technique, aiming to prevent the bias of the model toward low-severity data by balancing the amount of data in the two groups.

### Feature Selection and Deep Neural Network

To select the significant features influencing clinical severity, we evaluated the contribution of each of the 36 medical records (excluding gender information) on severity *via* feature importance analysis. For the feature importance values, we used the random forest (RF) (Breiman, 2001), extreme gradient boosting (XGBoost) (Chen and Guestrin, 2016), and adaptive boosting (AdaBoost) (Freund and Schapire, 1996; Ratsch et al., 2001) algorithms, which were the same as those in previous reports (Chung et al., 2021). After analyzing the feature importance values from each classifier algorithm, we normalized and averaged the values to determine the final feature importance values. By repeating the 5-fold cross-validation 10 times, we determined the best hyperparameters. For RF, we set the number of tree estimators to 100, the maximum depth to 4, and the maximum features to 5. For XGBoost, we set the maximum depth to 4, the learning rate to 0.1, and the number of tree estimators to 100. For AdaBoost, we set the number of tree estimators to 200, and the learning rate to 0.2.

Based on the ranked features, we applied them to a deep neural network (DNN) to predict the clinical severity. For the hyperparameters of the DNN, we investigated up to five hidden layers and each layer depth (node) up to the previous layer depth (node). For the input layer, we first ranked the features according to their final importance values, and then, evaluated the performance by increasing the number of top features in the input layer from 1 to 36. In the DNN, we also applied dropouts by changing the dropout rate from 0 to 0.5, with increments of 0.1. The last FC layer was fed into a sigmoid layer, which is an output layer providing the probabilities of patient severity. We trained the models using the ADAM optimizer and binary cross-entropy cost function with a learning rate of 0.0001 and batch size of 64. The models were implemented using R (version: 4.0.2) with TensorFlow (version 1.13.1) for DNN, scikit-learn (version 0.22.1) for machine learning algorithms, and xgboost (version 0.6.4) for the XGBoost algorithm.

For each set of top features, we found the best cross-validation accuracy using the two metrics [i.e., area under the curve (AUC) and balanced accuracy]. Balanced accuracy was calculated by averaging the sensitivity and specificity. Considering the cross-validation accuracy analysis, we finally modeled a four-layer DNN using the top 18 features for the male group and one using the top 10 features for the female group. For the male-dependent AI model, the input layer was fed into a series of three FC layers, comprising 18, 16, and 1 nodes. In the first two FC layers, we used a dropout rate of 0.3 and a leaky rectified linear unit (ReLU) activation with a slope of 0.2. For the female-dependent AI model, the input layer was fed into a series of three FC layers, comprising 10, 8, and 1 nodes. In the first two FC layers, we used a dropout rate of 0.3 and a leaky ReLU with a slope of 0.2.

## Results

### Feature Selection and Cross-Validation Results

**FIGURE 1:**
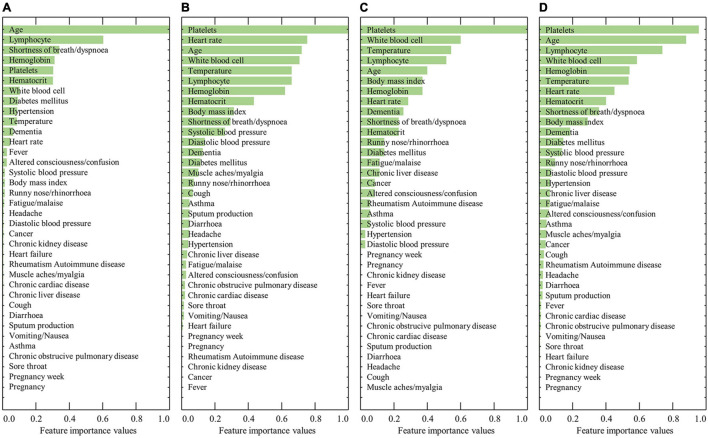
Results of the ranked feature importance values for the male group using **(A)** RF, **(B)** XGBoost, **(C)** AdaBoost, and **(D)** average after normalization.

Figure 1 shows the ranked feature importance from the male group using RF, XGBoost, AdaBoost, and a combination of the algorithms. The results from RF indicated that age had the highest importance value, followed by lymphocyte level, shortness of breath/dyspnea, hemoglobin, and platelets (Figure 1A). The results from XGBoost indicate that platelet count had the highest importance value, followed by heart rate, age, white blood cell count, and temperature (Figure 1B). The results from AdaBoost indicate that platelet count had the highest importance value, followed by white blood cell count, temperature, lymphocyte count, and age (Figure 1C). By averaging the values obtained from the three models, platelet count was determined to be the most important value, followed by age, lymphocyte count, white blood cell count, and hemoglobin level (Figure 1D). In contrast, previous comorbidities, such as diabetes mellitus, hypertension, heart failure, chronic cardiac disease, asthma, chronic obstructive pulmonary disease, chronic kidney disease, cancer, chronic liver disease, and rheumatism, rarely contributed to the predictive model. The detailed results of the feature importance value from RF, XGBoost, AdaBoost, and their combination are summarized in Supplementary Table 2.

**FIGURE 2:**
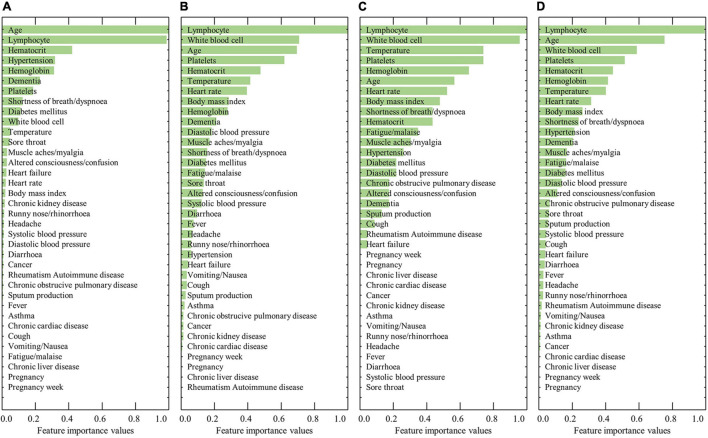
Results of the ranked feature importance values for the female group using **(A)** RF, **(B)** XGBoost, **(C)** AdaBoost, and **(D)** average after normalization.

Figure 2 shows the ranked feature importance from the female group using RF, XGBoost, AdaBoost, and a combination of the algorithms. The results from RF indicated that age had the highest importance value, followed by lymphocyte level, hematocrit, hypertension, and hemoglobin (Figure 2A). The results from XGBoost indicate that lymphocytes had the highest importance value, followed by white blood cell count, age, platelet count, and hematocrit (Figure 2B). The results from AdaBoost indicate that lymphocyte level had the highest importance value, followed by white blood cell count, temperature, platelet count, and hemoglobin (Figure 2C). By averaging the values obtained from the three models, lymphocyte level was determined to have the highest importance value, followed by age, white blood cell count, platelet count, and hematocrit (Figure 2D). The detailed results of the feature importance values from RF, XGBoost, AdaBoost, and their combination are summarized in Supplementary Table 3.

**FIGURE 3:**
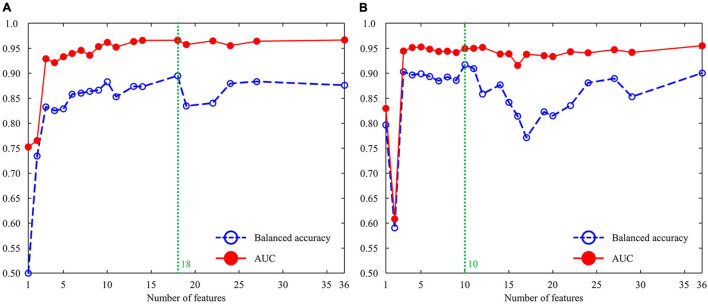
Cross-validation performance using the metrics of AUC and balanced accuracy: **(A)** male group and **(B)** female group. AUC, area under the curve.

We investigated the cross-validation performance using the metrics of AUC and balanced accuracy (Figure 3). For the male group (Figure 3A), both AUC and balanced accuracy reached the highest values when the top 18 features from the combination of AdaBoost, RF, and XGBoost were used as the input layer in the DNN. For the female group (Figure 3B), both AUC and balanced accuracy reached the highest values when the top 10 features from the combination of AdaBoost, RF, and XGBoost were used as the input layer in the DNN. Table 3 summarizes the cross-validation results for male- and female-dependent models. For the male group, the model provided a sensitivity of 0.83, specificity of 0.91, accuracy of 0.91, balanced accuracy of 0.87, and AUC of 0.96. For the female group, the model provided a sensitivity of 0.87, a specificity of 0.94, an accuracy of 0.94, a balanced accuracy of 0.91, and an AUC of 0.95.

**TABLE 3:** Cross-validation results (mean ± SD) from male- and female-group models.

**Male-group-based model**										
**Fold**	**Total**	**TN**	**FP**	**FN**	**TP**	**Sensitivity**	**Specificity**	**Accuracy**	**Balanced accuracy**	**AUC**

1	369	310	35	4	20	0.8333	0.8986	0.8943	0.8659	0.9266
2	370	314	31	5	20	0.8000	0.9101	0.9027	0.8551	0.9250
3	370	324	22	4	20	0.8333	0.9364	0.9297	0.8849	0.9458
4	370	326	21	3	20	0.8696	0.9395	0.9351	0.9045	0.9473
5	369	316	33	4	16	0.8000	0.9054	0.8997	0.8527	0.8930
Mean	1,848	318	28.4	4	19.2	0.83 ± 0.03	0.91 ± 0.02	0.91 ± 0.02	0.87 ± 0.02	0.93 ± 0.02

**Female-group-based model**										

**Fold**	**Total**	**TN**	**FP**	**FN**	**TP**	**Sen**	**Spe**	**Acc**	**BA**	**AUC**

1	526	456	51	1	18	0.9474	0.8994	0.9011	0.9234	0.9536
2	536	478	36	4	18	0.8182	0.9300	0.9254	0.8741	0.9398
3	526	491	8	3	24	0.8889	0.9840	0.9791	0.9364	0.9597
4	526	488	22	1	15	0.9375	0.9569	0.9563	0.9472	0.9648
5	518	466	39	3	10	0.7692	0.9228	0.9189	0.8460	0.9240
Mean	2,632	475.8	31.2	2.4	17	0.87 ± 0.07	0.94 ± 0.03	0.94 ± 0.03	0.91 ± 0.03	0.95 ± 0.01

*AUC, area under the curve.

*TN: true negatives, FP: false positives, FN: false negatives, TP: true positives.

### Testing Data Results

Table 4 summarizes the results of the test dataset. With the isolated test dataset, for the male group, the AI model using the four-layer DNN shows a sensitivity of 0.93, a specificity of 0.92, an accuracy of 0.92, a balanced accuracy of 0.93, and an AUC of 0.97. Similarly, for the female group, the AI model using the four-layer DNN shows a sensitivity of 0.93, a specificity of 0.93, an accuracy of 0.93, a balanced accuracy of 0.93, and an AUC of 0.95. The results were derived from the model trained by applying the male-group data to the male testing data only. In addition, the model trained by the female-group data was applied to the female testing data only.

**TABLE 4:** Testing data results from the same gender data.

**Male-dependent**									
**Model**	**TN**	**FP**	**FN**	**TP**	**Sensitivity**	**Specificity**	**Accuracy**	**Balanced accuracy**	**AUC**

Random forest	376	58	5	23	0.8214	0.8664	0.8636	0.8439	0.9236
XGBoost	402	32	8	20	0.7143	0.9263	0.9134	0.8203	0.9115
AdaBoost	401	33	4	24	0.8571	0.9240	0.9199	0.8906	0.9366
DNN	401	33	2	26	0.9286	0.9240	0.9242	0.9263	0.9660

**Female-dependent**									

**Model**	**TN**	**FP**	**FN**	**TP**	**Sensitivity**	**Specificity**	**Accuracy**	**Balanced accuracy**	**AUC**

Random forest	593	36	3	27	0.9000	0.9428	0.9408	0.9214	0.8596
XGBoost	612	17	8	22	0.7333	0.9730	0.9621	0.8532	0.8365
AdaBoost	559	70	2	28	0.9333	0.8887	0.8907	0.9110	0.8574
DNN	587	42	2	28	0.9333	0.9332	0.9332	0.9333	0.9539

*AUC, area under the curve; DNN, deep neural network.

*TN: true negatives, FP: false positives, FN: false negatives, TP: true positives.

To investigate the gender bias of each model, the model trained by the male-group data was applied to the female testing data only. Similarly, the model trained by the female-group data was applied to the male testing data only. Table 5 summarizes the results. First of all, when the model was trained by all the training data regardless of gender, the sensitivity, specificity, accuracy, balanced accuracy, and AUC were 0.97, 0.96, 0.96, 0.96, and 0.97 for the female testing data, respectively. For the male testing data, the sensitivity, specificity, accuracy, balanced accuracy, and AUC were 0.93, 0.94, 0.94, 0.93, and 0.98, respectively. However, when the model trained by the male-group data was applied to the female testing data only, the overall accuracy decreased—sensitivity from 0.93 to 0.86, specificity from 0.92 to 0.86, accuracy from 0.92 to 0.86, balanced accuracy from 0.93 to 0.86, and AUC from 0.97 to 0.94. For comparison, we investigated the female testing data on the unbiased model, which was trained using all the training data, regardless of gender. In the unbiased model, the overall accuracy increased, with sensitivity of 0.93, specificity of 0.94, accuracy of 0.94, balanced accuracy of 0.93, and AUC of 0.98. A bias tendency was also observed in the model trained using the female group only. When the model trained by the female-group data was applied to the male testing data, the overall accuracy also decreased—sensitivity from 0.97 to 0.90, specificity from 0.96 to 0.91, accuracy from 0.96 to 0.91, balanced accuracy from 0.96 to 0.90, and AUC from 0.97 to 0.95. Through these results, we confirmed that the unbiased model is superior to the biased model.

**TABLE 5:** Testing data results from different gender data.

**Testing data gender**	**Model**	**TN**	**FP**	**FN**	**TP**	**Sensitivity**	**Specificity**	**Accuracy**	**Balanced accuracy**	**AUC**
Female	Trained by all (unbiased)	605	24	1	29	0.9667	0.9618	0.9621	0.9643	0.9727
	Trained by male-group only (biased)	570	59	3	27	0.9000	0.9062	0.9059	0.9031	0.9499
	Random forest (biased)	536	93	2	28	0.9333	0.8521	0.8558	0.8927	0.9479
	XGBoost (biased)	611	18	8	22	0.7333	0.9714	0.9605	0.8524	0.9227
	AdaBoost (biased)	593	36	6	24	0.8000	0.9428	0.9363	0.8714	0.9495
Male	Trained by all (unbiased)	407	27	2	26	0.9286	0.9378	0.9372	0.9332	0.9795
	Trained by female-group only (biased)	375	59	4	24	0.8571	0.8641	0.8636	0.8606	0.9435
	Random forest (biased)	405	29	7	21	0.7500	0.9332	0.9221	0.8416	0.9338
	XGBoost (biased)	406	28	6	22	0.7857	0.9355	0.9264	0.8606	0.9398
	AdaBoost (biased)	374	60	4	24	0.8571	0.8618	0.8615	0.8597	0.9449

*AUC, area under the curve.

*TN: true negatives, FP: false positives, FN: false negatives, TP: true positives.

More importantly, when we evaluated each gender-dependent model with the test data from the same gender used for training, the resultant accuracy was also lower than that from the unbiased model. Regarding the accuracy, the male-dependent model and unbiased model provided 0.92 and 0.96 for the male-group test data, respectively. Similarly, the female-dependent model and unbiased model provided 0.93 and 0.94 for the female-group test data, respectively. These results indicate that training using only a specific group of data may limit the diversity of the entire data, so a model trained with only a specific group of data performs worse than an unbiased model, even on the specific group of data trained.

## Discussion and Conclusion

In this study, we investigated the model bias that can occur when training a model using only one particular gender dataset. For the bias investigation, we considered an AI model that predicts severity at an early stage based on the COVID-19 patient medical records. We trained and evaluated two separate models—one was trained using only the male group, and the other was trained with only the female group. The results showed that the gender-dependent AI model provided lower accuracy compared to the unbiased model. Furthermore, we found that the accuracy from the biased model was also lower than that from the unbiased model even with the test data from the same gender used for training. Despite using only male-group test data, the unbiased model provided higher accuracy than the male-dependent model. Similarly, despite using only female-group test data, the unbiased model provided higher accuracy than the female-dependent model. These results indicate that training using only a specific group of data limits the diversity of the entire data, and may provide lower accuracy even with the specific group of data trained. Supplementary Table 4 summarizes the normalized feature importance values from Adaboost, RF, and XGBoost, and the average when the model was trained with all training data regardless of gender. It shows that the gender feature was selected as the 16th highest value among the 37 medical records, indicating the gender information also affected the model to predict severity at an early stage of COVID-19 patients. Thus, it can be interpreted that the additional gender feature made a difference in accuracy compared to the biased model.

The ultimate vision of AI is the system that handles a wide range of cognitive tasks using a single general intelligence. However, such a general AI requires the same general capabilities as a human being, and most engineers have started focusing on more specific tasks that were more likely to be solved. Then, we may think that even a specific task would be better if it were further subdivided for a specific group of data (i.e., male-dependent model or female-dependent model). Our study began with this question, which was investigated through the COVID-19 issue, the topic of greatest interest to people around the world. Recently, some studies on AI bias according to gender have been conducted mainly in image recognition and natural language processing fields (Acien et al., 2018; Costa-jussà, 2019), but most AI studies have focused on improving performance through trial and error: hyperparameter search on networks (Forghani, 2020). Especially with the prediction model of severity or mortality at an early stage of COVID-19 (Altschul et al., 2020; Zhu et al., 2020; Lessmann et al., 2021; Paiva Proença Lobo Lopes et al., 2021; Shan et al., 2021; Yaşar et al., 2021), to our best knowledge, this paper serves as the first attempt to investigate the gender-specific models. Through this study, we found that a subdivided model for only a specific group of data had difficulty in overfitting issues due to the relatively small data sample size and the missing specific-group information (i.e., male or female). Regarding the sample size issue in our study, the male-dependent and female-dependent models were trained by 1,848 and 2,633 medical records, respectively. On the other hand, the unbiased model was trained by 4,480 medical records, which provided a better ability to generalize the model to new data regardless of gender. Thus, the bias in a subdivided model for only a specific group of data can be considered as an overfitting problem with limited data samples. We believe that this implication highlights the importance of generalizing to new data by maximizing the number of data samples rather than pursuing a granular model.

Our study has several limitations. First, we used a relatively small size of data (*n* = 5,601) for training and testing the models. This is because physicians are extremely busy fighting COVID-19, making it difficult to organize large amounts of data in this era of worldwide crisis. Thus, we will establish a sustainable AI training system that can keep training our model using prospectively collecting medical records. Then, we expect to minimize the overfitting issue originating from the small data sample size in a subdivided model for a specific group of data. Subsequently, we will revisit to investigate the subdivided model for specific gender data. Second, out of 5,601 patients, there were only nineteen pregnant women, which was 0.34% among all data and 0.57% among the female-group data. In addition, all of the pregnant women were in the low-severity group only. Due to the limited pool of pregnant women, the features of pregnancy and pregnancy weeks did not influence the feature selection process. Indeed, the pregnancy and the pregnancy week were with zero feature importance values for all methods (Supplementary Table 3). Thus, it might be necessary to update our AI model by training with larger and more diverse datasets. In addition to gender bias, academic and government officials have also raised concerns over racial bias in AI-based technologies (Parikh et al., 2019). In this study, we considered only the gender-dependent bias. In the near future, we plan to apply our AI models to cross-national datasets including data from patients of other races and investigate the bias study of race-dependent models. Addressing bias could allow AI to reach its fullest potential by helping to improve performance. Especially, in this COVID-19 pandemic, it is even more important to develop an unbiased AI model for diagnosis and prediction while protecting patients.

## Data Availability Statement

The original contributions presented in the study are included in the article/Supplementary Material, further inquiries can be directed to the corresponding author.

## Ethics Statement

The studies involving human participants were reviewed and approved by the Wonkwang University Hospital. Written informed consent for participation was not required for this study in accordance with the national legislation and the institutional requirements.

## Author Contributions

HC performed machine learning and deep learning simulations for hyperparameter search and modeling. CP and WK performed data validation for the COVID-19 patients. WK and JL validated and confirmed the simulations and helped draft the manuscript. JL conceived the study, participated in the study’s design and coordination, and wrote the initial manuscript. All authors read and approved the final manuscript.

## Conflict of Interest

The authors declare that the research was conducted in the absence of any commercial or financial relationships that could be construed as a potential conflict of interest.

## Publisher’s Note

All claims expressed in this article are solely those of the authors and do not necessarily represent those of their affiliated organizations, or those of the publisher, the editors and the reviewers. Any product that may be evaluated in this article, or claim that may be made by its manufacturer, is not guaranteed or endorsed by the publisher.
