# Supplementary material for: Gender Bias in Artificial Intelligence: Severity Prediction at an Early Stage of COVID-19
Source: Front Physiol. 2021 Nov 29;12:778720. doi: 10.3389/fphys.2021.778720 (PMC8667070; doi:10.3389/fphys.2021.778720)
Supplement: Supplementary file 1 [file Data_Sheet_1.docx]

Supplementary Material

# Supplementary Figures and Tables

## Supplementary Figures


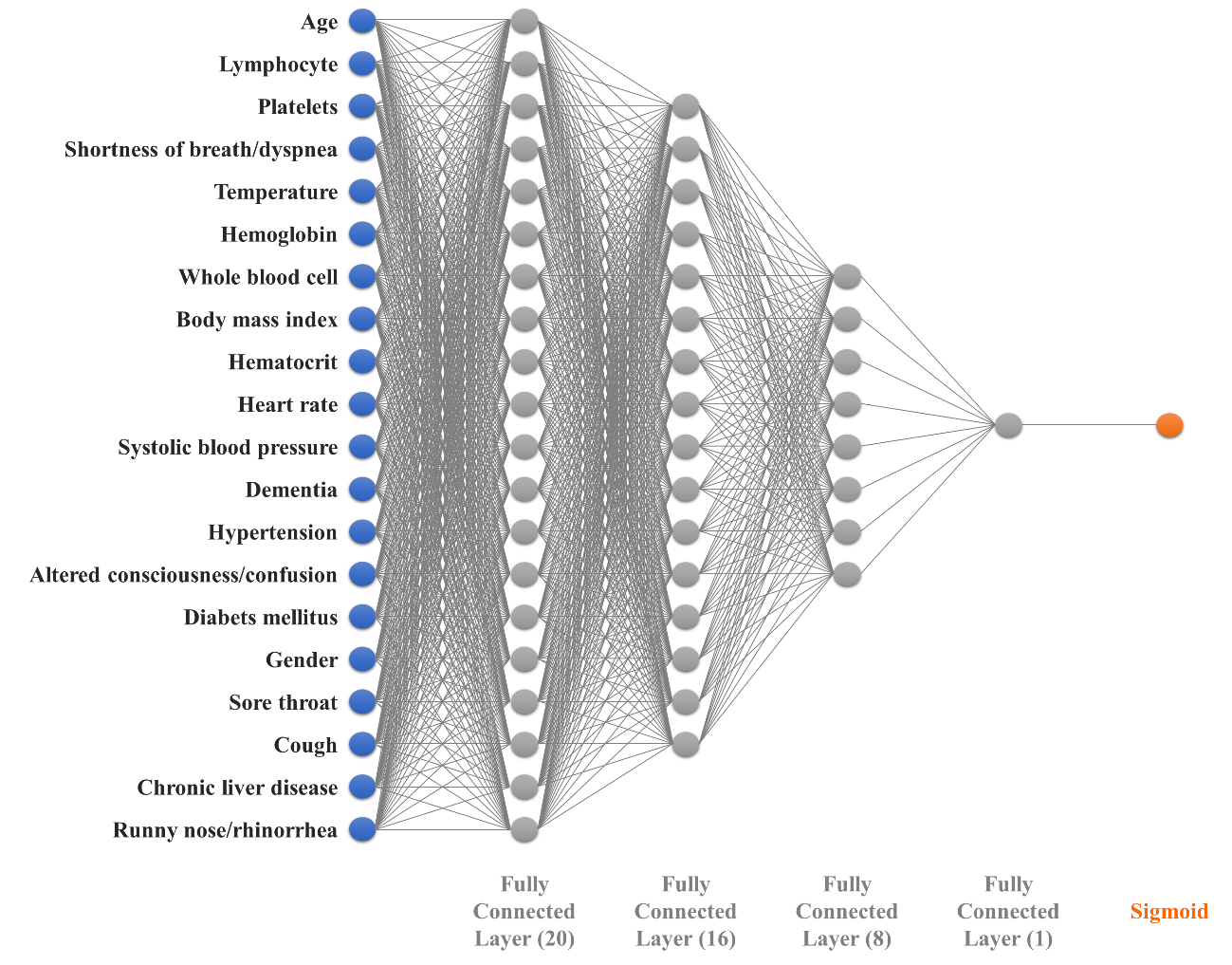


**Supplementary Figure 1**. Final artificial intelligence model with 5-layer deep neural network (DNN) for the COVID-19 severity prediction

## Supplementary Tables

**Supplementary Table 1**. Statistical summary of clinical features from the high-severity (*n*=271, 4.84%) and low-severity (*n*=5,330, 95.16%) groups (*n*=5,601)

| **No** | **Data** | | **Low-severity group (*n*=5,330)** | **High-severity group (*n*=271)** | ***p*** |
| --- | --- | --- | --- | --- | --- |
| Basic patient information | | | | | |
| **1** | Age | | 4.26 ± 1.92 | 7.05 ± 1.08 | <0.001 |
|  |  |  | 0) 0-9, 1) 10-19, 2) 20-29, 3) 30-39, 4) 40-49,  5) 50-59, 6) 60-69, 7) 70-79, 8) 80- | |  |
| **2** | Gender | Male | 2,166 | 144 | <0.001 |
|  |  | Female | 3,164 | 127 |  |
| **3** | Pregnancy | | 19 | 0 | 0.3259 |
| **4** | Pregnancy week | | 16.50 ± 10.01 | - | - |
| Physical index | | | | | |
| **5** | Body mass index | | 1.79 ± 1.02 | 1.84 ± 1.13 | 0.5416 |
|  |  |  | 0) <18.5, 1)18.5-22.9, 2) 23.0-24.9, 3) 25.0-29.9, 4) >30 | |  |
| Initial examination findings | | | | | |
| **6** | Systolic blood pressure | | 1.75 ± 1.31 | 1.98 ± 1.46 | 0.0078 |
|  |  |  | 0) <120, 1) 120-129, 2) 130-139, 3) 140-159, 4) >160 | |  |
| **7** | Diastolic blood pressure | | 1.00 ± 0.97 | 0.90 ± 1.00 | 0.1090 |
|  |  |  | 0) <80, 1) 80-89, 2) 90-99, 3) >100 | |  |
| **8** | Heart rate | | 85.66 ± 14.79 | 89.05 ± 19.64 | <0.001 |
| **9** | Temperature | | 36.94 ± 0.54 | 37.11 ± 0.80 | <0.001 |
| Clinical findings | | | | | |
| **10** | Fever | | 1197/5326 (22%) | 105/271 (39%) | <0.001 |
| **11** | Cough | | 2239/5326 (42%) | 92/271 (34%) | 0.0084 |
| **12** | Sputum production | | 1532/5326 (29%) | 79/271 (29%) | 0.8909 |
| **13** | Sore throat | | 858/5326 (16%) | 14/271 (5%) | <0.001 |
| **14** | Runny nose/rhinorrhoea | | 609/5326 (11%) | 8/271 (3%) | <0.001 |
| **15** | Muscle aches/myalgia | | 894/5326 (17%) | 26/271 (10%) | 0.0018 |
| **16** | Fatigue/malaise | | 215/5326 (4%) | 18/271 (7%) | 0.0362 |
| **17** | Shortness of breath/dyspnoea | | 531/5326 (10%) | 134/271 (49%) | <0.001 |
| **18** | Headache | | 946/5326 (18%) | 17/271 (6%) | <0.001 |
| **19** | Altered consciousness/confusion | | 9/5326 (0%) | 26/271 (10%) | <0.001 |
| **20** | Vomiting/Nausea | | 226/5326 (4%) | 18/271 (7%) | 0.0592 |
| **21** | Diarrhoea | | 496/5326 (9%) | 20/271 (7%) | 0.2834 |
| Current or previous comorbidity diseases | | | | | |
| **22** | Diabetes mellitus | | 582/5327 (11%) | 106/271 (39%) | <0.001 |
| **23** | Hypertension | | 1034/5327 (19%) | 164/271 (61%) | <0.001 |
| **24** | Heart failure | | 39/5327 (1%) | 20/271 (7%) | <0.001 |
| **25** | Chronic cardiac disease | | 150/5311 (3%) | 29/271 (11%) | <0.001 |
| **26** | Asthma | | 115/5327 (2%) | 13/271 (5%) | 0.0046 |
| **27** | Chronic obstructive pulmonary disease | | 31/5327 (1%) | 9/271 (3%) | <0.001 |
| **28** | Chronic kidney disease | | 37/5327 (1%) | 18/271 (7%) | <0.001 |
| **29** | Cancer | | 123/5326 (2%) | 22/271 (8%) | <0.001 |
| **30** | Chronic liver disease | | 76/5004 (2%) | 7/271 (3%) | 0.1704 |
| **31** | Rheumatism/ Autoimmune diseases | | 35/4998 (1%) | 3/271 (1%) | 0.4410 |
| **32** | Dementia | | 148/5001 (3%) | 76/271 (28%) | <0.001 |
| General blood test results | | | | | |
| **33** | Haemoglobin | | 13.37 ± 1.69 | 11.89 ± 2.23 | <0.001 |
| **34** | Haematocrit | | 39.51 ± 4.72 | 35.28 ± 6.56 | <0.001 |
| **35** | Lymphocyte | | 30.08 ± 11.12 | 15.08 ± 10.69 | <0.001 |
| **36** | Platelets | | 239.96 ± 81.57 | 188.51 ± 87.38 | <0.001 |
| **37** | White blood cell | | 6.00 ± 2.55 | 7.99 ± 5.10 | <0.001 |

**Supplementary Table 2**. Normalized feature importance from Adaboost, RF and XGBoost, and the ranked feature importance with those average (Male)

| **No** | **Features** | **RF** | **XGBoost** | **AdaBoost** | **Three models** |
| --- | --- | --- | --- | --- | --- |
| 1 | Platelets | 0.3033 | 1.0000 | 1.0000 | 0.7678 |
| 2 | Age | 1.0000 | 0.7200 | 0.4000 | 0.7067 |
| 3 | Lymphocyte | 0.6032 | 0.6600 | 0.5143 | 0.5925 |
| 4 | White blood cell | 0.1030 | 0.7067 | 0.6000 | 0.4699 |
| 5 | Hemoglobin | 0.3113 | 0.6200 | 0.3714 | 0.4342 |
| 6 | Temperature | 0.0862 | 0.6600 | 0.5429 | 0.4297 |
| 7 | Heart rate | 0.0459 | 0.7533 | 0.2857 | 0.3616 |
| 8 | Hematocrit | 0.3018 | 0.4333 | 0.2286 | 0.3212 |
| 9 | Shortness of breath/dyspnoea | 0.3397 | 0.2933 | 0.2286 | 0.2872 |
| 10 | Body mass index | 0.0132 | 0.3133 | 0.3714 | 0.2327 |
| 11 | Dementia | 0.0649 | 0.1267 | 0.2571 | 0.1496 |
| 12 | Diabetes mellitus | 0.0915 | 0.1133 | 0.1429 | 0.1159 |
| 13 | Systolic blood pressure | 0.0134 | 0.2600 | 0.0571 | 0.1102 |
| 14 | Runny nose/rhinorrhoea | 0.0125 | 0.0733 | 0.1429 | 0.0762 |
| 15 | Diastolic blood pressure | 0.0066 | 0.1400 | 0.0286 | 0.0584 |
| 16 | Hypertension | 0.0874 | 0.0400 | 0.0286 | 0.0520 |
| 17 | Chronic liver disease | 0.0042 | 0.0333 | 0.1143 | 0.0506 |
| 18 | Fatigue/malaise | 0.0095 | 0.0267 | 0.1143 | 0.0501 |
| 19 | Altered consciousness/confusion | 0.0258 | 0.0267 | 0.0857 | 0.0460 |
| 20 | Muscle aches/myalgia | 0.0045 | 0.1000 | 0.0000 | 0.0348 |
| 21 | Asthma | 0.0005 | 0.0467 | 0.0571 | 0.0348 |
| 22 | Cancer | 0.0066 | 0.0000 | 0.0857 | 0.0308 |
| 23 | Cough | 0.0030 | 0.0667 | 0.0000 | 0.0232 |
| 24 | Rheumatism Autoimmune disease | 0.0046 | 0.0000 | 0.0571 | 0.0206 |
| 25 | Headache | 0.0067 | 0.0467 | 0.0000 | 0.0178 |
| 26 | Diarrhoea | 0.0028 | 0.0467 | 0.0000 | 0.0165 |
| 27 | Sputum production | 0.0018 | 0.0467 | 0.0000 | 0.0161 |
| 28 | Fever | 0.0265 | 0.0000 | 0.0000 | 0.0088 |
| 29 | Chronic cardiac disease | 0.0043 | 0.0200 | 0.0000 | 0.0081 |
| 30 | Chronic obstrucive pulmonary disease | 0.0005 | 0.0200 | 0.0000 | 0.0068 |
| 31 | Vomiting/Nausea | 0.0008 | 0.0133 | 0.0000 | 0.0047 |
| 32 | Sore throat | 0.0003 | 0.0133 | 0.0000 | 0.0045 |
| 33 | Heart failure | 0.0046 | 0.0067 | 0.0000 | 0.0037 |
| 34 | Chronic kidney disease | 0.0057 | 0.0000 | 0.0000 | 0.0019 |
| 35 | Pregnancy | 0.0000 | 0.0000 | 0.0000 | 0.0000 |
| 36 | Pregnancy week | 0.0000 | 0.0000 | 0.0000 | 0.0000 |

**Supplementary Table 3**. Normalized feature importance from Adaboost, RF and XGBoost, and the ranked feature importance with those average (Female)

| **No** | **Features** | **RF** | **XGBoost** | **AdaBoost** | **Three models** |
| --- | --- | --- | --- | --- | --- |
| 1 | Lymphocyte | 0.9865 | 1.0000 | 1.0000 | 0.9955 |
| 2 | Age | 1.0000 | 0.6939 | 0.5652 | 0.7530 |
| 3 | White blood cell | 0.0974 | 0.7075 | 0.9565 | 0.5871 |
| 4 | Platelets | 0.1861 | 0.6190 | 0.7391 | 0.5147 |
| 5 | Hematocrit | 0.4201 | 0.4762 | 0.4348 | 0.4437 |
| 6 | Hemoglobin | 0.3114 | 0.2789 | 0.6522 | 0.4141 |
| 7 | Temperature | 0.0484 | 0.4150 | 0.7391 | 0.4008 |
| 8 | Heart rate | 0.0218 | 0.3946 | 0.5217 | 0.3127 |
| 9 | Body mass index | 0.0192 | 0.2857 | 0.4783 | 0.2611 |
| 10 | Shortness of breath/dyspnoea | 0.1232 | 0.1565 | 0.4348 | 0.2382 |
| 11 | Hypertension | 0.3173 | 0.0612 | 0.2609 | 0.2131 |
| 12 | Dementia | 0.2267 | 0.2109 | 0.1739 | 0.2038 |
| 13 | Muscle aches/myalgia | 0.0294 | 0.1701 | 0.3043 | 0.1679 |
| 14 | Fatigue/malaise | 0.0008 | 0.1429 | 0.3478 | 0.1638 |
| 15 | Diabetes mellitus | 0.1107 | 0.1497 | 0.2174 | 0.1593 |
| 16 | Diastolic blood pressure | 0.0080 | 0.1837 | 0.2174 | 0.1364 |
| 17 | Altered consciousness/confusion | 0.0284 | 0.1293 | 0.1739 | 0.1105 |
| 18 | Chronic obstrucive pulmonary disease | 0.0059 | 0.0136 | 0.1739 | 0.0645 |
| 19 | Sore throat | 0.0442 | 0.1361 | 0.0000 | 0.0601 |
| 20 | Sputum production | 0.0056 | 0.0272 | 0.1304 | 0.0544 |
| 21 | Systolic blood pressure | 0.0103 | 0.1224 | 0.0000 | 0.0442 |
| 22 | Cough | 0.0025 | 0.0340 | 0.0870 | 0.0412 |
| 23 | Heart failure | 0.0255 | 0.0408 | 0.0435 | 0.0366 |
| 24 | Diarrhoea | 0.0076 | 0.0952 | 0.0000 | 0.0343 |
| 25 | Fever | 0.0048 | 0.0748 | 0.0000 | 0.0266 |
| 26 | Headache | 0.0104 | 0.0680 | 0.0000 | 0.0261 |
| 27 | Runny nose/rhinorrhoea | 0.0121 | 0.0612 | 0.0000 | 0.0245 |
| 28 | Rheumatism Autoimmune disease | 0.0059 | 0.0000 | 0.0435 | 0.0164 |
| 29 | Vomiting/Nausea | 0.0020 | 0.0340 | 0.0000 | 0.0120 |
| 30 | Chronic kidney disease | 0.0156 | 0.0136 | 0.0000 | 0.0098 |
| 31 | Asthma | 0.0035 | 0.0204 | 0.0000 | 0.0080 |
| 32 | Cancer | 0.0074 | 0.0136 | 0.0000 | 0.0070 |
| 33 | Chronic cardiac disease | 0.0026 | 0.0068 | 0.0000 | 0.0031 |
| 34 | Chronic liver disease | 0.0006 | 0.0000 | 0.0000 | 0.0002 |
| 35 | Pregnancy | 0.0001 | 0.0000 | 0.0000 | 0.0000 |
| 36 | Pregnancy week | 0.0000 | 0.0000 | 0.0000 | 0.0000 |

**Supplementary Table 4.**  Normalized feature importance from Adaboost, RF and XGBoost, and the ranked feature importance with those average (Both male and female)

| **No** | **Features** | **RF** | **XGBoost** | **AdaBoost** | **Three models** |
| --- | --- | --- | --- | --- | --- |
| 1 | Age | 1.00 | 0.96 | 0.86 | 0.94 |
| 2 | LYMPHO | 0.75 | 0.95 | 0.89 | 0.86 |
| 3 | PLT | 0.25 | 1.00 | 1.00 | 0.75 |
| 4 | SOB | 0.39 | 0.41 | 0.51 | 0.44 |
| 5 | Temperature | 0.03 | 0.67 | 0.55 | 0.42 |
| 6 | HGB | 0.16 | 0.55 | 0.51 | 0.41 |
| 7 | WBC | 0.08 | 0.67 | 0.33 | 0.36 |
| 8 | BMI | 0.02 | 0.37 | 0.61 | 0.33 |
| 9 | HCT | 0.24 | 0.44 | 0.18 | 0.29 |
| 10 | HR | 0.02 | 0.63 | 0.18 | 0.28 |
| 11 | SBP | 0.01 | 0.35 | 0.32 | 0.22 |
| 12 | DEMEN | 0.17 | 0.20 | 0.30 | 0.22 |
| 13 | HTN | 0.21 | 0.11 | 0.15 | 0.16 |
| 14 | ACC | 0.04 | 0.22 | 0.22 | 0.16 |
| 15 | DM | 0.09 | 0.15 | 0.21 | 0.15 |
| 16 | Gender | 0.01 | 0.17 | 0.18 | 0.12 |
| 17 | Cough | 0.00 | 0.07 | 0.16 | 0.08 |
| 18 | ST | 0.02 | 0.11 | 0.10 | 0.08 |
| 19 | CLD | 0.00 | 0.11 | 0.13 | 0.08 |
| 20 | RNR | 0.01 | 0.09 | 0.09 | 0.06 |
| 21 | DBP | 0.01 | 0.17 | 0.01 | 0.06 |
| 22 | HEADA | 0.01 | 0.06 | 0.05 | 0.04 |
| 23 | MAM | 0.00 | 0.07 | 0.04 | 0.04 |
| 24 | Fever | 0.01 | 0.07 | 0.00 | 0.03 |
| 25 | CKD | 0.01 | 0.04 | 0.02 | 0.02 |
| 26 | DIARR | 0.00 | 0.04 | 0.00 | 0.01 |
| 27 | VN | 0.00 | 0.02 | 0.00 | 0.01 |
| 28 | Cancer | 0.00 | 0.01 | 0.00 | 0.00 |
| 29 | FM | 0.00 | 0.01 | 0.00 | 0.00 |
| 30 | COPD | 0.00 | 0.01 | 0.00 | 0.00 |
| 31 | SPUTUM | 0.00 | 0.01 | 0.00 | 0.00 |
| 32 | CCD | 0.00 | 0.01 | 0.00 | 0.00 |
| 33 | HF | 0.00 | 0.00 | 0.00 | 0.00 |
| 34 | Asthma | 0.00 | 0.00 | 0.00 | 0.00 |
| 35 | RDAD | 0.00 | 0.00 | 0.00 | 0.00 |
| 36 | Pregnancy | 0.00 | 0.00 | 0.00 | 0.00 |
| 37 | Pregnancy week | 0.00 | 0.00 | 0.00 | 0.00 |
